# Glaucoma Rehabilitation using ElectricAI Transcranial Stimulation (GREAT)—study protocol for randomized controlled trial using combined perceptual learning and transcranial electrical stimulation for vision enhancement

**DOI:** 10.1186/s13063-024-08314-3

**Published:** 2024-07-22

**Authors:** Shuwen Jia, Xiaolin Mei, Lilin Chen, Lok Hin Chan, Celia Tsang, Venus Suen, Tingni Li, Myo Win Zaw, Amanda Liu, Ben Thompson, Bernhard Sabel, George Woo, Christopher K. S. Leung, Shea-ping Yip, Dorita H. F. Chang, Allen M. Y. Cheong

**Affiliations:** 1 School of Optometry, The Hong Kong Polytechnic University, Hong Kong Special Administrative Region, China; 2 Centre for Eye and Vision Research Limited, Hong Kong Science Park, Hong Kong Special Administrative Region, China; 3 School of Optometry and Vision Science, University of Waterloo, Waterloo, Canada; 4 Institute of Medical Psychology, University of Magdeburg, Magdeburg, Germany; 5 Department of Ophthalmology, The University of Hong Kong, Hong Kong Special Administrative Region, China; 6 Department of Health Technology and Informatics, The Hong Kong Polytechnic University, Hong Kong Special Administrative Region, China; 7 Department of Psychology, The University of Hong Kong, Hong Kong Special Administrative Region, China; 8 Research Centre for SHARP Vision, The Hong Kong Polytechnic University, Hong Kong Special Administrative Region, China

**Keywords:** Glaucoma, Vision rehabilitation, Visual field, High-resolution perimetry, Quality of life, Transcranial electrical stimulation, Perceptual learning, Mental health, Mobility

## Abstract

**Background:**

Glaucoma patients with irreversible visual field loss often experience decreased quality of life, impaired mobility, and mental health challenges. Perceptual learning (PL) and transcranial electrical stimulation (tES) have emerged as promising interventions for vision rehabilitation, showing potential in restoring residual visual functions. The Glaucoma Rehabilitation using ElectricAI Transcranial stimulation (GREAT) project aims to investigate whether combining PL and tES is more effective than using either method alone in maximizing the visual function of glaucoma patients. Additionally, the study will assess the impact of these interventions on brain neural activity, blood biomarkers, mobility, mental health, quality of life, and fear of falling.

**Methods:**

The study employs a three-arm, double-blind, randomized, superiority-controlled design. Participants are randomly allocated in a 1:1:1 ratio to one of three groups receiving: (1) real PL and real tES, (2) real PL and sham tES, and (3) placebo PL and sham tES. Each participant undergoes 10 sessions per block (~ 1 h each), with a total of three blocks. Assessments are conducted at six time points: baseline, interim 1, interim 2, post-intervention, 1-month post-intervention, and 2-month post-intervention. The primary outcome is the mean deviation of the 24-2 visual field measured by the Humphrey visual field analyzer. Secondary outcomes include detection rate in the suprathreshold visual field, balance and gait functions, and electrophysiological and biological responses. This study also investigates changes in neurotransmitter metabolism, biomarkers, self-perceived quality of life, and psychological status before and after the intervention.

**Discussion:**

The GREAT project is the first study to assess the effectiveness of PL and tES in the rehabilitation of glaucoma. Our findings will offer comprehensive assessments of the impact of these treatments on a wide range of brain and vision-related metrics including visual field, neural activity, biomarkers, mobility, mental health, fear of falling, and quality of life.

**Trial registration:**

ClinicalTrials.gov NCT05874258. Registered on May 15, 2023.

**Supplementary Information:**

The online version contains supplementary material available at 10.1186/s13063-024-08314-3.

## Administrative information

Note: the numbers in curly brackets in this protocol refer to the SPIRIT checklist item numbers. The order of the items has been modified to group similar items (see http://www.equator-network.org/reporting-guidelines/spirit-2013-statement-defining-standard-protocol-items-for-clinical-trials/).

**Table:** 

Title {1}	Glaucoma Rehabilitation using ElectricAI Transcranial Stimulation (GREAT)—study protocol for randomized controlled trial using combined perceptual learning and transcranial electrical stimulation for vision enhancement
Trial registration {2a and 2b}	ClinicalTrials.gov; NCT05874258; 15th May 2023 https://clinicaltrials.gov/study/NCT05874258?cond=NCT05874258&rank=1
Protocol version {3}	Version 2, 28th June 2024
Funding {4}	Hong Kong Research Grants Council Research Impact Fund (RIF R5047-19) The Hong Kong Special Administrative Region Government and InnoHK The Hong Kong Polytechnic University Research Postgraduate Scholarship
Author details {5a}	Shuwen Jia^a#^, Xiaolin Mei^a#^, Lilin Chen^a^, Lok Hin Chan^a^, Celia Tsang^a^, Venus Suen^a^, Tingni Li^b^, Myo Win Zaw^b^, Amanda Liu^b^, Ben Thompson^b,c^, Bernhard Sabel^d^, George Woo^a^, Christopher K. S. Leung^e^, Shea-ping Yip^f^, Dorita H. F. Chang^g^ and Allen M. Y. Cheong^a,b,h^* Affiliations: ^a^School of Optometry, The Hong Kong Polytechnic University, HKSAR; ^b^Centre for Eye and Vision Research Limited, Hong Kong Science Park, HKSAR; ^c^School of Optometry and Vision Science, University of Waterloo, Canada; ^d^Institute of Medical Psychology, University of Magdeburg, Germany; ^e^Department of Ophthalmology, The University of Hong Kong, HKSAR; ^f^Department of Health Technology and Informatics, The Hong Kong Polytechnic University, HKSAR; ^g^Department of Psychology, The University of Hong Kong, HKSAR; ^h^Research Centre for SHARP Vision, The Hong Kong Polytechnic University, HKSAR ^#^These two authors contributed equally to this work *Corresponding author
Name and contact information for the trial sponsor {5b}	Allen M. Y. Cheong (principal investigator), School of Optometry, The Hong Kong Polytechnic University, HKSAR (allen.my.cheong@polyu.edu.hk)
Role of sponsor {5c}	This is an investigator-initiated clinical trial. The sponsor and funders played no role in the design of the study, data collection, analysis, interpretation of data, and writing the manuscript.

## Introduction

### Background and rationale {6a}

Glaucoma, an optic neuropathy that results in visual field loss [1], has a profound impact on an individual’s quality of life (QoL) [2], mental health [3], and mobility [4]. In Hong Kong, glaucoma accounted for 10% of visual impairment cases [5]. Various pharmaceutical treatments are available to reduce intraocular pressure and slow the progression of glaucoma [6]. However, the damage to vision caused by glaucoma is irreversible, profoundly affecting daily life. Vision rehabilitation can complement medical treatments by helping glaucoma patients maximize the use of their remaining vision, thereby preserving or recovering functional vision. Certain visual rehabilitation strategies, including the use of optical devices, accessible technologies, and techniques to maximize vision (eccentric viewing), have proven beneficial for reading, mobility, and QoL [7–9]. However, the effectiveness and adoption of these approaches are often hindered by factors like low public acceptance and deterioration of visual functions [10]. Besides, issues related to device usability or psychological factors such as frustration when using the devices can increase the abandonment rate of rehabilitation devices [11]. Hence, there is a growing interest in exploring new approaches to vision rehabilitation that focus on retraining the brain to enhance the neural processing of residual visual information from the retina.

Given that vision loss does not typically result in complete blindness, there is potential for improving residual visual function through reactivating damaged but surviving retinal cells and enhancing the processing of information from healthy cells, a concept known as neuroplasticity [12]. Neuroplasticity provides an optimized view of rehabilitation. While the mechanisms of neuroplasticity in vision science are not fully understood, two principles might be involved [13]: (1) residual visual activation [12] and (2) modulation of the brain’s functional connectivity networks [14]. Emerging evidence has demonstrated that vision can be improved by harnessing neuroplasticity [15–17]. The most commonly employed approaches for leveraging neuroplasticity are perceptual learning (PL) [18, 19] and transcranial electrical stimulation (tES) [20, 21].

PL refers to improved performance of a visual task with repeated practice or training [22, 23]. PL can enhance performance across a wide range of visual tasks including vernier acuity, contrast sensitivity, and motion direction discrimination [22, 24, 25]. The mechanisms underlying PL include altered tuning of neural populations and a gradual reweighting of inputs to perceptual decision-making networks [26]. The application of PL to glaucoma patients is still at an early stage, although initial clinical trial results have been reported. For example, Sabel and Gudlin [27] observed that 3 months of daily training (6 days per week for 1 h per day) significantly improved detection accuracy and reaction time measured using high-resolution perimetry (HRP) compared to a placebo. However, the absence of interim assessments throughout the long duration of intensive training has obscured the dose-response relationship. The confirmation of a dose-response relationship would provide stronger evidence supporting the practical application of PL in glaucoma rehabilitation.

tES is a non-invasive brain stimulation technique that can modify the excitability and synchronization of targeted neural regions and networks. The stimulation is achieved by delivering a mild electric current through electrodes mounted on the head that alters ongoing brain activity [28]. There is a growing body of evidence suggesting that tES could be a useful tool for vision rehabilitation [29, 30]. For example, tES has been found to improve vision in a wide range of clinical conditions, including age-related macular degeneration [31, 32], retinitis pigmentosa [33], amblyopia [20, 34], and hemianopia [35, 36]. Furthermore, results from our phase 1 study in the GREAT (Glaucoma Rehabilitation using ElectricAI Transcranial stimulation—NCT04846140) revealed that a single session of anodal transcranial direct current stimulation (tDCS) enhanced perceptual and electrophysiological measures of vision in patients with glaucoma [37]. Besides, tES is a non-invasive technique that is generally safe and typically associated with only mild, transient discomfort [38, 39]. Therefore, the potential application of tES as a tool for vision rehabilitation in glaucoma patients is promising.

### Objectives {7}

#### Specific objectives

The aims of this study are to (1) compare the effects of monotherapy (either PL or tES) and combined therapy (PL + tES) on improving visual field function; (2) examine whether the interventions influence brain neural activity, and concentrations of neurotransmitters in the brain and blood; (3) investigate the degree to which enhancements in visual field or brain neural activity, brought about by the interventions, act as moderating factors in the improvement of mobility performance, mental health, fear of falling, and QoL; and (4) optimize the training protocol and the optimal dosage for achieving maximal intervention effect in glaucoma patients.

Hypotheses:

- The combination of PL and tES will result in greater visual field improvements than monotherapy.
- The intervention effect will increase with longer training duration.
- The improvement in the visual field induced by the interventions will be accompanied by changes in brain activities and blood biomarkers.
- An improved visual field will contribute to better balance function, gait performance, and QoL. It will also decrease fear of falling and symptoms of depression or anxiety in glaucoma patients.

### Trial design {8}

**Fig. 1:**
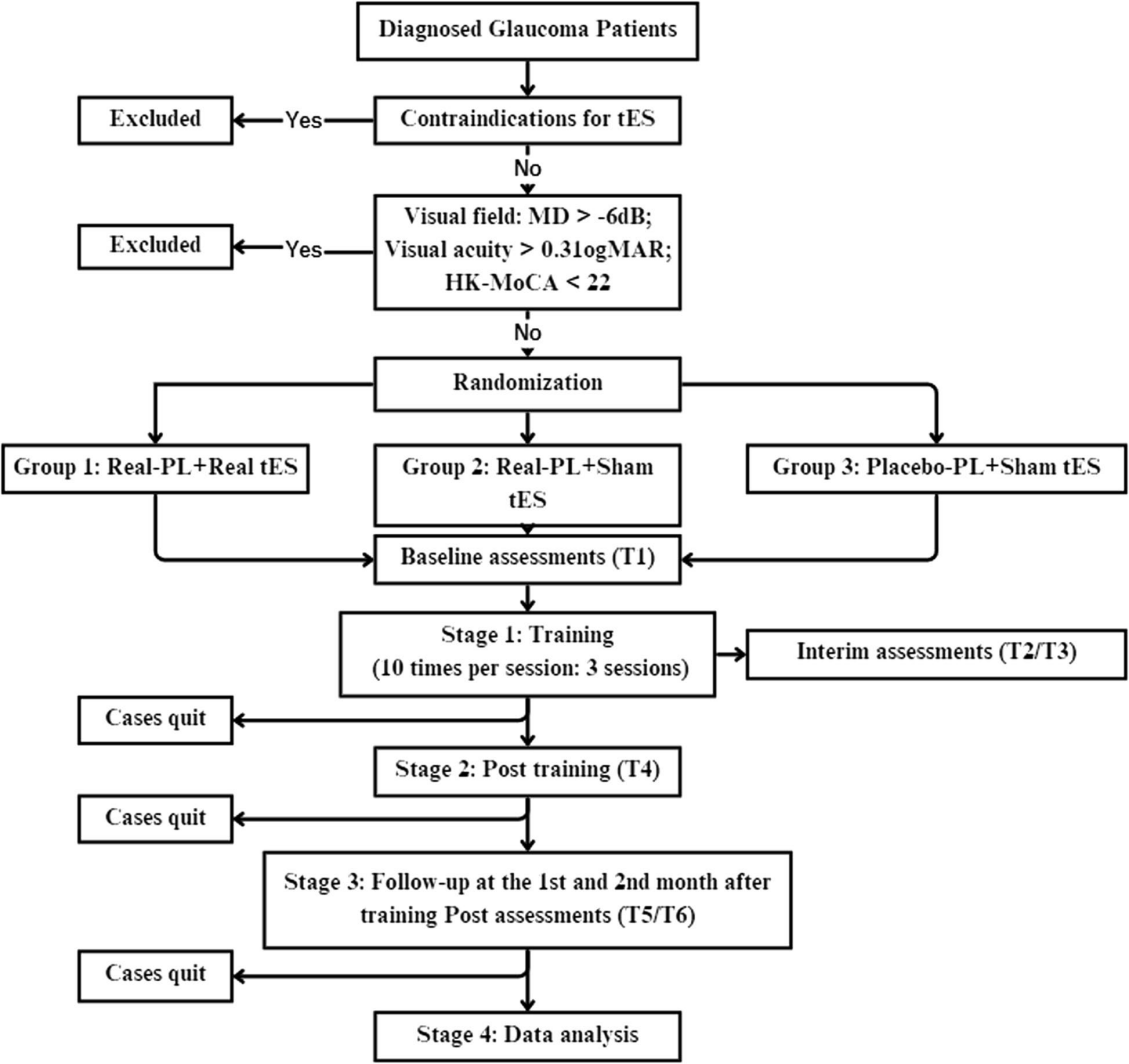
Flow chart for the study

This protocol is designed for a three-arm, double-blind, randomized, superiority-controlled design with 1:1:1 allocation. Figure 1 shows the flow chart of this study.

## Methods: participants, interventions, and outcomes

### Study setting {9}

The research study is conducted at two locations in Hong Kong SAR—The Hong Kong Polytechnic University and Grantham Hospital. The Hong Kong Polytechnic University serves as the primary site for all detailed assessments, both before and after the intervention. The university’s well-equipped laboratories and facilities ensure precise and comprehensive data collection, providing a robust foundation for the research. To enhance accessibility and convenience for participants, the training sessions are held at either The Hong Kong Polytechnic University or Grantham Hospital, depending on which location is more suitable for each individual. Grantham Hospital is a public general hospital located in a different area of Hong Kong, allowing the study to reach a broader range of participants across different districts.

### Eligibility criteria {10}

All participants who give their consent undergo a phone eligibility screening. During the screening, they are asked about their medical history, medication history, current eye disease conditions, and overall health status. Those who are potentially eligible are then screened for ocular health, cognitive function, and physical function based on the following inclusion and exclusion criteria.

#### Inclusion criteria

- Age from 18 to 80 years
- Diagnosis of primary open-angle or normal-tension glaucoma with relative scotoma in both eyes
- Stable vision for at least 3 months
- Absence of ocular diseases other than glaucoma
- Humphrey visual field analyzer (HFA) visual field loss (mean deviation of ≤ − 6 dB) within the central 24° of the visual field for both eyes
- Best-corrected distance visual acuity (BCVA) of 6/12 (equivalent to 0.3 logMAR) or better for binocular vision and the eye with better visual field
- A cognitive functional score of 22 or above in the Montreal Cognitive Assessment—Hong Kong version (HK-MoCA) [40]

#### Exclusion criteria

- Ocular diseases other than glaucoma (e.g., age-related macular degeneration, diabetic retinopathy, moderate to severe cataract) or severe hearing impairment (to ensure that participants can hear the instructions clearly during assessments and training)
- Severe medical problems (e.g., stroke, Parkinson’s disease) or self-reported neurological (e.g., brain surgery, brain tumor, peripheral neuropathy), or cognitive disorders (e.g., diagnosed dementia or cognitive impairment)
- Self-reported vestibular or cerebellar dysfunction, history of vertigo
- Use of medications for neurological or psychiatric conditions that might interfere with motor control
- Contraindications for tES, including:
  - History of adverse reaction to tES
  - History of seizure (epilepsy) in self or a first-degree relative
  - History of frequent or severe headaches
  - History of head injury, intracranial surgery
  - Pericranial or intracranial metallic objects (e.g., shrapnel, surgical clips, screws, or wires)
  - History of any neurological disorder (e.g., encephalitis, meningitis, stroke, brain tumor)
  - Advanced, unstable, or uncontrolled medical condition (e.g., recent myocardial infarction, pneumonia, end-stage renal or hepatic failure, poorly controlled diabetes)
  - Pregnancy; sexually active and not using a reliable method of birth control
  - Use of illicit drugs
  - Significant alcohol intake (> 2 standard drinks) or sleep deprivation (much less the usual) in the last 24 h
  - Any skin disorder affecting the head or face

### Who will take informed consent? {26a}

This study adheres to the latest version of the Declaration of Helsinki. A trained research assistant obtains informed consent from the participants by providing detailed explanation of the project when they complete the consent form. Informed consent must be obtained before any examinations or questionnaires administered.

### Additional consent provisions for collection and use of participant data and biological specimens {26b}

The blood test and magnetic resonance spectroscopy (MRS) are optional measures. The request for blood sample collection is covered in the original informed consent procedure and an additional informed consent is provided to participants who are willing and eligible for the MRS measure.

## Interventions

### Explanation for the choice of comparators {6b}

Although both PL and tES have shown beneficial effects in restoring visual functions in patients with glaucoma, it is still unclear whether a combined approach can offer greater benefits than monotherapy. Therefore, our goal is to compare the effectiveness of combined PL and tES with monotherapy in improving the visual field in glaucoma patients. Through this comparative study, we aim to understand the potential combined effects and identify the optimal approach for enhancing visual rehabilitation in glaucoma patients.

### Intervention description {11a}

The intervention comprises of PL and tES. Depending on the group they are assigned to, participants receive one of the different combinations of perceptual training and stimulation. The eye with the least visual field defect will be chosen as the training eye, a choice made to maximize the potential benefits of the training intervention in the patients’ daily lives. In the rare instances when the visual field defects are identical in both eyes, the eye with higher visual acuity is chosen as the training eye.

For PL, a customized computer-based program specifically designed for vision restoration training will be utilized. The selection of a region of interest (ROI) is a crucial step, which is based on neighborhood weighting and eccentricity factors derived from the baseline visual field of high-resolution perimetry (HRP). To mitigate random responses observed during HRP testing, a neighborhood weighting rule is applied, where the accuracy value of each point is replaced by the average value of its surrounding 20 points. Additionally, the weighting of points is influenced by their eccentricity, giving higher importance to central points compared to peripheral ones. This process generates a map with varying accuracy scales, from which the 40 most promising training points are selected within the ROI. Within this ROI, a Gabor discrimination task is employed as the training task, with the training difficulty adjusted through contrast variations (initially set at 0.8 and ending at 0.1). The Gabor size is m-scaled according to eccentricity, and two Gabor orientations (horizontal or vertical) are presented to the training positions of the training eye monocularly.

In the real PL group, 80% of the training positions are selected from the ROI, while the remaining 20% are selected from the central 5-degree region. Conversely, in the placebo PL group, 80% of the training positions are chosen from the central 5-degree area, and the remaining 20% are selected from the ROI.

A 1-up and 1-down session-by-session staircase strategy is used for each session. If the accuracy of a given position falls within the 75 to 85% range, the contrast remains the same for subsequent training sessions. If the accuracy drops below 75%, the difficulty is decreased by increasing the contrast in increments of 0.1. Conversely, if the accuracy exceeds 85%, the difficulty is increased by reducing the contrast by 0.1. Once the visual performance (discrimination accuracy) of a training position shows significant improvement and stability for three consecutive sessions, the training position expands radially (by 1 degree) into the surrounding area to enhance the visual training effect. The entire training procedure is automatically controlled by a customized computer program, following the aforementioned rules. The difficulty level for each session is determined based on the participant’s performance in the previous session. To evaluate the effectiveness of the training, the experimenter monitors the participant’s results on a weekly basis.

tES will be administered using a direct current stimulation (tDCS) protocol with a Nurostym tES device (Neuro Device Group SA, Poland). tDCS is a commonly used protocol that may increase the excitability of stimulated cells and affect neuronal resting membrane potentials, thereby altering the local concentration of neurotransmitters [41, 42]. Stimulation is delivered by two 5 cm × 5 cm rubber electrodes placed inside saline-soaked sponges, with the current intensity set to 2 mA, as per previous studies [43, 44]. Participants receive either active anodal tDCS or sham anodal tDCS for 20 min, with 20 s of fade-in and fade-out, depending on their assigned group. The anodal electrode is positioned at Oz (visual cortex), while the cathodal electrode is placed on the cheek. The choice of cheek side is determined by the trained glaucoma eye and the location of the visual field defect to enhance the stimulation effect. For instance, if the left eye is the training eye with a more severe visual defect in the left field, the right cheek will be positioned as the cathodal site. In cases where there is no obvious lateralized injury in the training eye, the contralateral cheek of the training eye will be selected as the cathodal site.

Participants will be randomly assigned to three different groups.

1. Real PL + real tES. In this group, participants will receive 30 training sessions (10-time training × 3 blocks) with real PL and real tES (2 ~ 3 sessions per week, about 1 h per session).
2. Real PL + sham tES. In this group, participants will receive 30 training sessions (10-time training × 3 blocks) with real PL and sham tES (2 ~ 3 sessions per week, about 1 h per session).
3. Placebo PL + sham tES. In this group, participants will receive 30 training sessions (10-time training × 3 blocks) with placebo PL and sham tES (2 ~ 3 sessions per week, about 1 h per session).

### Criteria for discontinuing or modifying allocated interventions {11b}

Given that the procedures in the current study are generally low-risk, the likelihood of participants’ withdrawal or discontinuation is minimal. However, since the majority of our participants are elderly individuals, we will closely monitor and promptly address any discomfort or changes in their health that are unrelated to the study that may affect their participation.

### Strategies to improve adherence to interventions {11c}

To improve study adherence, each participant receives personalized attention from a trained research staff member on the training day and during all assessments. Participants are encouraged to stay focused and approach the entire training procedure with confidence. These measures are designed to promote engagement and commitment to the study interventions.

### Relevant concomitant care permitted or prohibited during the trial {11d}

During recruitment and prior to each treatment session, participants are instructed not to participate in any concurrent interventions throughout the duration of the study. This includes other non-invasive brain stimulation experiments or any forms of physical training. All urgent interventions or treatments that occur will be documented in the research records.

### Provisions for post-trial care {30}

No provision for post-trial care will be provided, as the interventions have been found to be safe. Participants will return to the standard care when the trials conclude. Besides, all enrolled participants will be provided with insurance, which provides compensation for any study-related injuries.

### Outcomes {12}

**Fig. 2:**
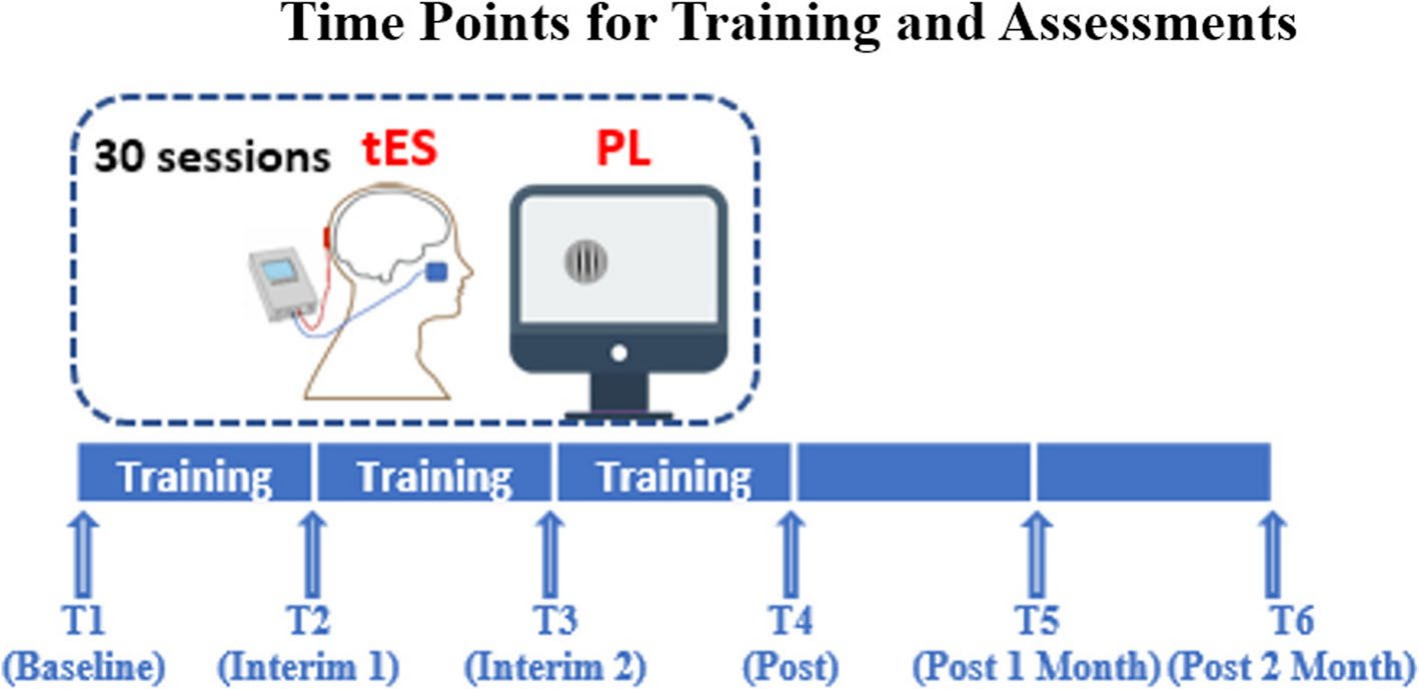
The six time points for the 30-session trainings (PL + tES) and the assessments

**Fig. 3:**
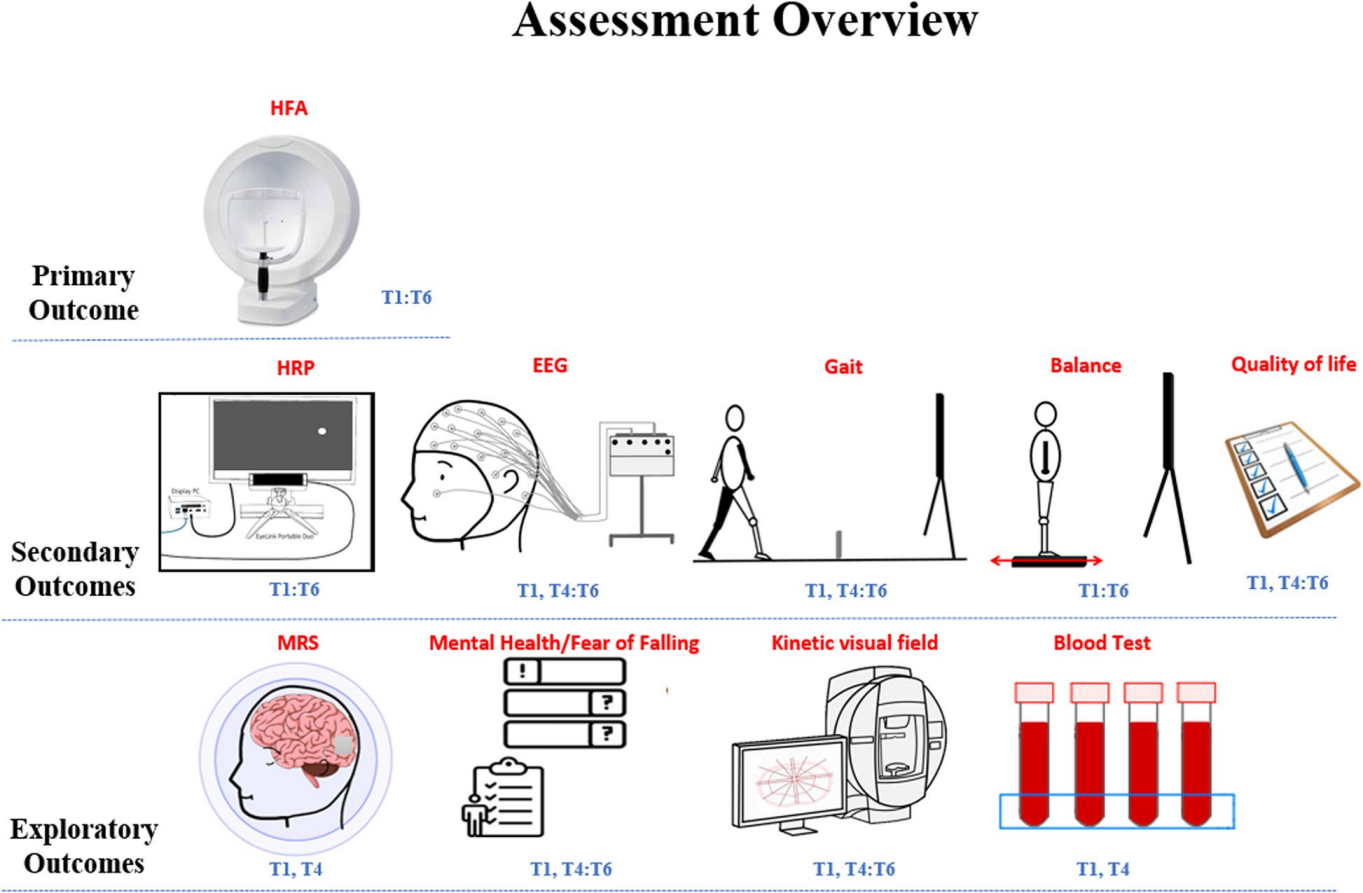
Assessment overview: this figure provides a comprehensive view of all assessments conducted during the current clinical trial and the time points of each assessment

Participants will have thirty-session trainings, with assessments conducted at six different time points. These assessments consist of a baseline test (T1), a test after completing the first block of training (T2), a test after completing the second block of training (T3), a test after completing all training sessions (T4), a test 1 month after completing all training sessions (T5), and a test 2 months after completing all training sessions (T6). The time points for training and assessment can be seen from Fig. 2. At the baseline, participants’ ocular health will be assessed, including (1) visual acuity (VA): tested monocularly and binocularly using the Early Treatment of Diabetic Retinopathy Study (ETDRS) chart with best-corrected refractive corrections and habitual spectacle corrections; (2) contrast sensitivity (CS): assessed using a MARS Numerical Contrast Sensitivity at 50 cm (with appropriate near addition); and (3) retinal nerve fiber layer thickness (RNFL) using spectralis optical coherence tomography (OCT, Heidelberg Engineering, Heidelberg, Germany). Outcomes that will be repeatedly assessed throughout the entire experiment procedure are shown in Fig. 3.

#### Primary outcome

Visual field test is measured monocularly using the 24–2 and 10–2 Swedish interactive threshold algorithm (SITA) standard tests by Humphrey visual field analyzer (HFA, Carl Zeiss Meditec Inc., California). The mean deviation (MD), pattern standard deviation (PSD), and visual field index (VFI) are recorded and the MD of 24–2 visual field test is used as primary outcome of intervention effectiveness. HFA visual field test is conducted at six time points, from T1 to T6.

#### Secondary outcomes

1. HRP

The current HRP is a valid and reliable computer-based visual field assessment based upon a previously well-established program [45]. The revised HRP uses circular geometry instead of a rectangle to present stimuli, while maintaining its high-resolution advantage. During the HRP test, suprathreshold stimuli are presented in a radial pattern within 20 degrees, with a step size of 3 degrees. These stimuli are presented monocularly at a total of 98 positions, with the order of presentation randomized. To ensure a stable result and accurate assessment of participants’ responses, the HRP test is repeated five times. Throughout the HRP test, fixation is monitored by an infrared eye tracker (SR Research, Eyelink Portable Duo). HRP testing is conducted at six time points, from T1 to T6.

2) Electroencephalography (EEG)

EEG is used to reveal the pathological and neural mechanisms of intervention. Three task-related EEG experiments are designed to detect changes in integrated and peripheral visual function brought by the training. Additionally, the resting state EEG is recorded to reveal any changes in functional connectivity changes induced by training. A high-resolution 64-channel Quik-Cap Neo Net from Compumedics Neuroscan, in conjunction with a SynAmps amplifier, will be employed for EEG recording. During the process, the impedance from all channels will be maintained below 10 kΩ.

The ISCEV standard visual evoked potentials (VEP) protocol serves as the first EEG task [46]. A 1 Hz (500 ms in each phase) pattern reversal checkerboard is presented to the full visual field, and participants are asked to fixate on a red central point to maintain fixation. To improve the repeatability, the stimuli will be presented in two blocks of 34 s (68 reversals per block). The sequence of recordings is left eye first, followed by the right eye, and then both eyes during each EEG visit. The event-related potential (ERP) components derived from the task, such as the peak amplitude, peak latency, and peak latency/amplitude of N75, P100, and N135, will be analyzed.

The second task involves a 6 Hz steady-state visual evoked potential experiment (SSVEP) with each block lasting 20 s and a total of 3 blocks. Compared to VEP, the SSVEP has a higher signal-to-noise ratio (SNR) and may be more suitable for detecting changes in participants with severe glaucoma. A Fourier transformation will be used to extract the 6 Hz induced amplitude response. Additionally, the averaging method of neighborhood frequencies will be used to calculate the SNR.

The third task involves a modified version of radial flashed SSVEP. Instead of using the traditional rectangle reversal checkerboard, this design incorporates 5 differently sized embedded rings. The dartboard design is based on multifocal visual evoked potential (mfVEP) stimulus design [47]. In the experiment, the rings will flash at a frequency of 6 Hz in random order to elicit peripheral responses. Each ring will flash for three blocks, with each block lasting 20 s. Consequently, the induced amplitude and SNR at 6 Hz will be calculated as the measures of intervention.

For the resting-state EEG, 3-min closed eye and 3-min open eye will be recorded. To increase participants’ compliance, soft music of a same sound track is played during the resting state recording at each visit. During the eyes-open recording, participants are asked to fixate a physical hourglass to help minimize eye movement [48]. Measures of the intervention effect will include peak alpha, spectrum power, and functional connectivity network. EEG tests are conducted at four time points—T1, T4 to T6 (refer to Table 2 for details).

3) Balance and gait tests

Patients with visual field loss reported a higher risk of falling compared with those with normal vision [49] and balance and gait performance are significant factors in falls among glaucoma patients [50]. Therefore, improving balance and/or gait performance can be an important predictor of QoL improvement and safety among patients with glaucoma.

**Fig. 4:**
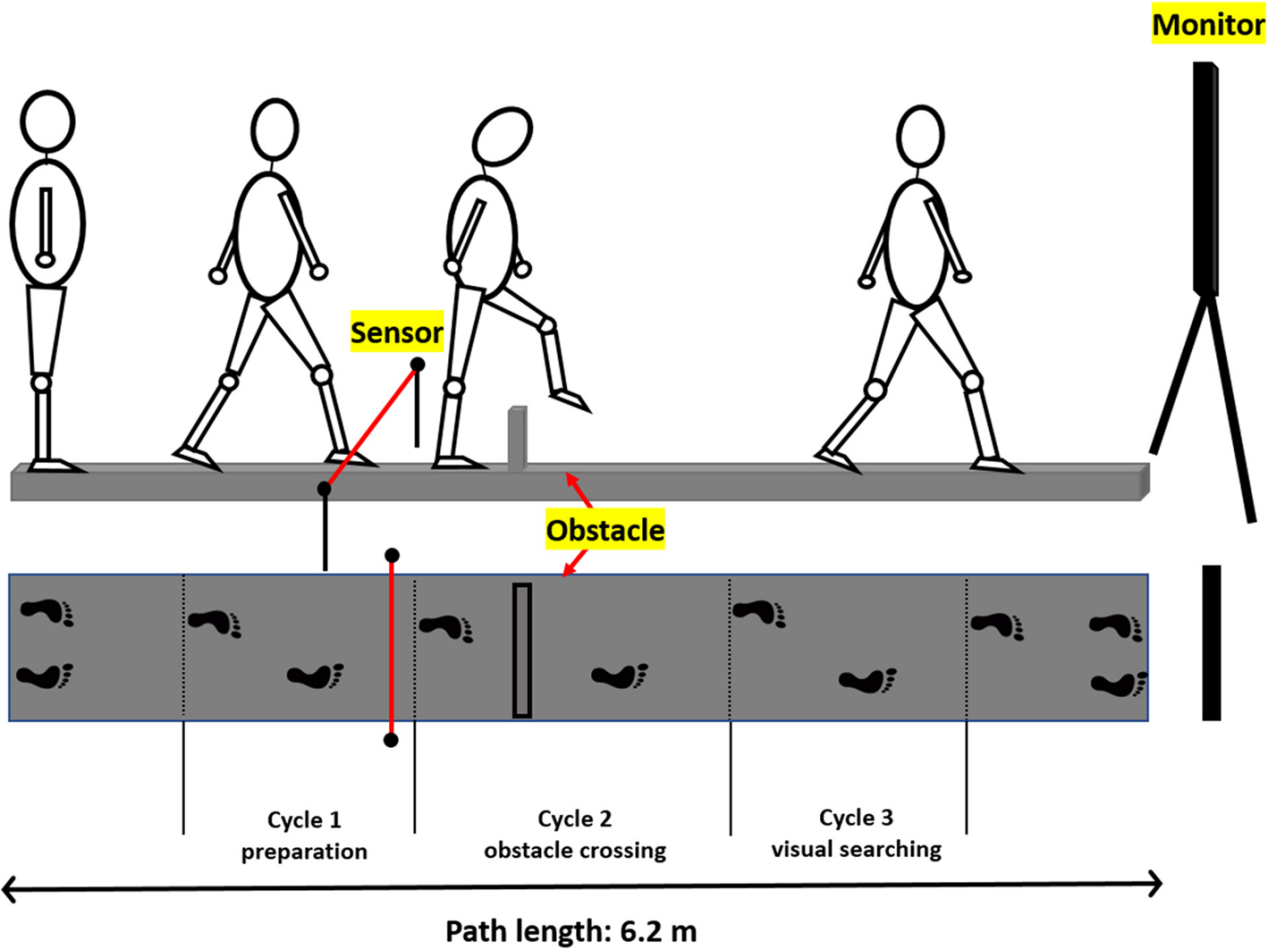
Pictorial presentation of the gait test: participants are asked to walk, cross an obstacle, and perform a visual search task presenting in the monitor while walking. Data from three consecutive gait cycles (i.e., two footsteps per cycle) are collected and analyzed, including one cycle before obstacle crossing, one cycle while crossing the obstacle (or stepping onto a marked position for the no-obstacle condition), and another cycle after crossing the obstacle. To accommodate participants with different walking speed, the visual stimuli will be presented after he/she walks pass the infrared sensor which is located at 1.5 m before the obstacle

- Gait test (Fig. 4)

Participants’ gait performance is recorded using the Vicon Motion System (Vicon Nexus 2.11, Oxford, UK). Twenty-seven reflective markers are placed on the head, trunk, and lower body according to the Vicon Plug-in full body gait model. All participants are asked to walk barefoot along a 6.2-m walkway at their normal comfortable towards a monitor at the end of the walkway, which presents a visual search task. Two impediments are introduced during the walk: physical obstacles and a visual search task. The obstacles can be gray for low contrast or yellow for high contrast, and either 5 or 15 cm in height. The base of the obstacle is 2.5 × 60 cm and positioned in the middle of the pathway. Participants’ gait parameters are measured under these conditions: (1) with or without an obstacle (5 sub-conditions: no obstacle; 5 cm gray obstacle; 5 cm yellow obstacle; 15 cm gray obstacle; 15 cm yellow obstacle) and (2) with or without a visual task (2 sub-conditions: fixation vs. Chinese character search). Five repetitions for each condition are recorded. Data from three consecutive gait cycles are collected and analyzed, including one cycle before obstacle crossing, one cycle while crossing the obstacle (or stepping onto a marked position for the no obstacle condition), and another cycle after crossing the obstacle (1 preparation, 1 obstacle crossing, 1 visual search). Gait parameters of the dominant leg are measured at 4 time points (T1, T4, T5, and T6) for each of the three cycles. These parameters include hip flexion/extension (the minimum/maximum angle in degrees), knee flexion/extension (the minimum/maximum angle in degrees), ankle flexion/extension (the minimum/maximum angle in degrees), head down/up (the minimum/maximum angle in degrees), walking speed (mm/s), stride length (mm), swing phase (%), and visual task accuracy (Appendix 1).

- Balance test

The Bertec Balance Advantage™ system (Bertec Corporation, Columbus, OH, USA) is used to assess participants’ balance before and after training. During the test, participants are asked to stand under different challenging conditions. Balance-challenging conditions include (1) standing on a firm or foam surface (Fi/Fo); (2) standing surface with backward or forward translation (BT/FT); and (3) standing with their eyes close (EC). Cognitively-challenging conditions include a visual search task (fixation vs. Chinese character search; see below). Standing surface and translation conditions are combined with a randomly presented visual task and allocated to participants. Besides, additional EC conditions are set for participants when they stand on the firm and foam surfaces to calculate the contribution of vision to postural control. Participants are required to repeat each condition three times (Table 1). Outcome measures, including root mean square sway of the center of pressure in the anterior–posterior and medial–lateral directions (mm/s), total sway path length (mm), maximum CoP displacement in the AP and ML directions (mm), latency reaction to translation (ms), visual task accuracy (%), and the contribution of vision to balance from eye close to eye open (%), are collected at six time points, from T1 to T6.

**Table 1:** Measurement conditions for balance test

**Conditions**		**Standing surface**	**Visual task**
**Translation**	Backward	Firm	Fixation
			Character searching
		Foam	Fixation
			Character searching
	Forward	Firm	Fixation
			Character searching
		Foam	Fixation
			Character searching
**Eye condition**	Eyes close	Firm	–
		Foam	–

**Fig. 5:**
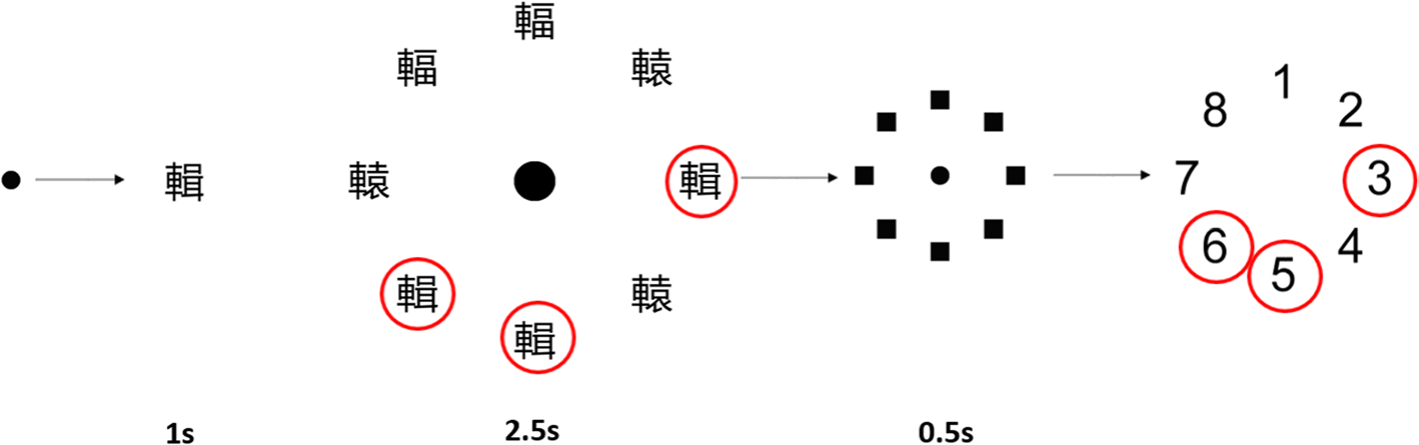
Visual search task included in balance and gait test. Participants are instructed to fixate on the central dot which then changes to a “target” Chinese character “輯” for 1 s. Subsequently, an array of 8 Chinese characters, including 3 targets, are the presented for 2.5 s. After this, a mask is presented for 0.5 s. Participants are then asked to report the positions of the targets

- Visual task (Fig. 5)

A visual search task is included in the balance and gait tests. In this task, 8 Chinese characters arranged in a circle are presented on the screen. Some characters are targets, while others are distractors which resemble the target characters. The visual task is run using MATLAB 2020b (The MathWorks Inc, Natick, MA). Nighty eight groups of Chinese characters, each character’s number of strokes ranging from 10 to 25, are prepared for the task. All characters are presented in “Microsoft JhengHei” font on either a 14″ monitor (for balance test) or a 46″ monitor (for gait test). Each character has an angular subtense of one degree (equivalent to a visual acuity of 6/360) and is randomly presented at one of the eight positions in a circular array with an angular subtense of eight degrees. In each trial, targets and distractors are randomly positioned. Participants are asked to identify the locations of target characters among the distractors during their balance or gait test and report the locations at the end of the test. The characters, displayed in white on a black background, are placed 1 m away from the force platform in the balance test and at the endpoint of the gait path for the gait test. Before the trial, participants are asked to fixate on a black spot presented on the screen. When the experiment starts, the target character replaces the fixation spot for 1 s. This is followed by the display of an array of 8 characters for 2.5 s, a masking screen for 0.5 s (to eliminate the after-image). Participants are instructed to respond by touching the screen to indicate the locations and numbers of target characters when they are close to the monitor. The accuracy of the response is recorded and analyzed.

4) Quality of life (QoL)

The ultimate objective of glaucoma rehabilitation is to sustain and enhance the QoL for patients. Therefore, it is important to evaluate whether the interventions employed in this study significantly improve QoL in glaucoma patients. The Chinese version of the National Eye Institute 25-Item Visual Function Questionnaire (NEI-VEQ-25) [51] and the Chinese version of the Low Vision Quality of Life Questionnaire (CLVQOL) [52] are used to assess vision-related QoL at four time points—T1, T4 to T6.

The NEI-VEQ-25 encompasses 12 domains: general health, general vision, ocular pain, near activities, distance activities, social functioning, mental health, role difficulties, dependency, driving, color vision, and peripheral vision [53]. Most individual items are scored by respondents using a 5- or 6-point response scale, ranging from (1) “not affected at all” to (5) “stopped doing this because of my eyesight” or (6) “stopped doing this for other reasons.” True/false items are scored on a 5-point response scale, ranging from (1) “definitely true” to (5) “definitely false,” with (3) indicating “not sure.” Responses for each item are converted to a score between 0 and 100, with higher scores representing better visual functioning [54].

The CLVQOL is a commonly used tool to examine general vision-related quality-of-life. It contains 25 closed-ended ordinal scale items including general vision and lighting, mobility, psychological adjustment, reading, fine work, and activities of daily living [52, 55]. Each item is scored on a scale of 5 (no problem due to vision) to 1 (great difficulty due to vision), with the total score being the sum of the item scores, ranging from 25 to 125. A lower total score indicates greater difficulty in performing daily life activities due to low vision.

#### Exploratory outcomes

1. Magnetic resonance spectroscopy (MRS)

Magnetic resonance spectroscopy (MRS) is a non-invasive imaging technique that enables the measurement of the chemical composition of brain tissues. The objective of this measure is to explore the neural mechanisms that underpin visual rehabilitation by examining changes in metabolite levels. Previous research has demonstrated the role of (gamma-aminobutyric acid) GABA + in inhibitory control, while glutamate/glutamine (Glx) primarily affects excitatory control, thereby contributing to the balance between inhibition and excitation (known as the I-E balance). A recent study showed that both GABA and glutamate level was associated with glaucoma severity and targeting GABA could possibly enhance the neural specificity in visual cortex [56]. To obtain accurate metabolic concentrations, the MEGA-PRESS sequence will be used to calculate GABA + values, and the PRESS sequence will be used to measure Glx levels.

Only individuals who are physically capable will be invited to undergo the MRS scan. Participants will be required to attend two magnetic resonance imaging (MRI) sessions: one before the training intervention and another after the training (T1 and T4). Before arriving at the MRI center, the experimental procedure will be explained, and participants will complete an additional consent form for the scanning. They will also be instructed to avoid consuming caffeine and alcohol for at least 24 h prior to the scan.

The scanning procedure begins with the acquisition of a high-resolution T1-weighted image using the MPRAGE sequence, with the following parameters: TR/TE/TI: 2500/2.13/1120 ms, flip angle: 8°, bandwidth: 220 Hz, voxel size: 0.8 × 0.8 × 0.8 mm^3^, using a 64-channel head coil. An experienced radiographer will position the volume of interest (VOI) in the primary visual cortex (V1). Following this, a single-voxel MRS using the MEGA-PRESS sequence with a TE of 68 ms and TR of 1500 ms will be performed. The scan, with a voxel size of 30 × 30 × 30 mm^3^ and 160 scan averages, will be preceded by 16 averages of water reference and will take approximately 9 min. Subsequently, the PRESS sequence with a TR/TE of 3000/30 and 128 averages will be used, lasting about 5 min. This scan will also be preceded by 8 averages of water reference. Finally, a resting-state scan with a TR/TE of 2000/30 will be conducted to measure any changes in functional connectivity. To ensure consistent recordings, a photograph will be taken at each instance to document the position of V1. This will help that subsequent scans for the same participants are conducted in the exact same location.

As the MRS technique is relatively new and the analysis methods are still under development, we will employ Osprey to fit the GABA + values and GANET for the Glx values. These software tools are utilized for the precise quantification and analysis of the metabolite values derived from the MRS data. For the resting state fMRI data, a seed-based functional connectivity analysis will be employed to examine the correlation between the visual cortex and other brain regions both before and after the intervention (T1 and T4). Additionally, a network-based analysis will be used to identify any other functional changes associated with the intervention. These analysis methods will provide valuable insights into the relationships and potential alterations within the brain following the intervention.

2) Mental health

Chronic diseases like glaucoma come with comorbid conditions such as depression or anxiety which can significantly decrease treatment adherence [57]. In this study, participants’ mental health is assessed using the Chinese version of the Patient Health Questionnaire-9 (PHQ-9) [58] and the Chinese version of the Perceived Stress Scale (PSS-10) [59, 60] at four time points—T1, T4 to T6. Both of them have demonstrated good validity and reliability. The PHQ-9 is a 9-item depression module, with each item scored from 0 (not at all) to 3 (nearly every day). Similarly, the PSS-10 consists of 10 items, each scored from 1 to 4. A higher total score on either scale indicates more severe depression or stress.

3) Fear of falling

Falling is a significant concern for glaucoma patients, with the fear of falling being a major contributing factor to the incidence of falls [61]. Moreover, fear of falling is associated with a decreased physical and social activity [62]. The Chinese version of the Falls Efficacy Scale-International (FES-I) is a validated and reliable questionnaire used to assess fear of falling in everyday life [63, 64]. This questionnaire includes sixteen items that are related to common daily activities, each graded on a scale of from 1 (not at all concerned) to 4 (very concerned). Higher scores indicate a greater concern about falling, which may indicate a poorer balance ability [63]. This questionnaire is conducted at four time points—T1, T4 to T6.

4) Kinetic visual field

The kinetic test is highly sensitive in detecting changes in the far peripheral visual field, which significantly correlates with balance function and gait performance [65, 66]. The binocular kinetic visual field is measured using Octopus 900 perimetry (Haag-Streit AG, Switzerland). Tests are done in the kinetic mode using the standard protocol (sixteen vectors), with a stimulus size of III4e moving at a speed of 5 degrees/s to map the hill of vision. Any changes in the area of the isopter over time would indicate the effect of training on the kinetic visual field [67]. Kinetic visual field is conducted at four time points—T1, T4 to T6.

5) Blood test

Emerging evidence has underscored the role of neurotrophin “brain-derived neurotrophic factor” (BDNF) in controlling synaptic plasticity (e.g., improved motor skills [68] and memory [69] through BDNF secretion after anodal tDCS). Besides, a high prevalence of anxiety or depression has been observed in patients with glaucoma [70]. Cortisol, a key component of the physiological stress response, is a commonly used stress biomarker [71]. Therefore, changes in serum BDNF concentration and cortisol concentration induced by intervention will be measured before and after the training (T1 and T4). The gene that regulates BNDF varies among individuals and this genetic polymorphism can influence neural plasticity [72]. To examine the relationship between BDNF Val66Met polymorphisms (rs6265 > A) and changes in visual function following intervention, genetic analysis of BDNF Val66Met polymorphisms will be conducted before the intervention (T1).

After obtaining participants’ informed consent, a volume of 6 mL blood will be collected. Serum plasma will be analyzed using an enzyme-linked immunosorbent assay (ELISA) for blood serum BDNF and cortisol [73]. DNA will be extracted from the leucocytes for the determination of BDNF Val66Met polymorphism using a method based on polymerase chain reaction (PCR).

### Participant timeline {13}

The timeline is shown in Table 2.

**Table 2:**
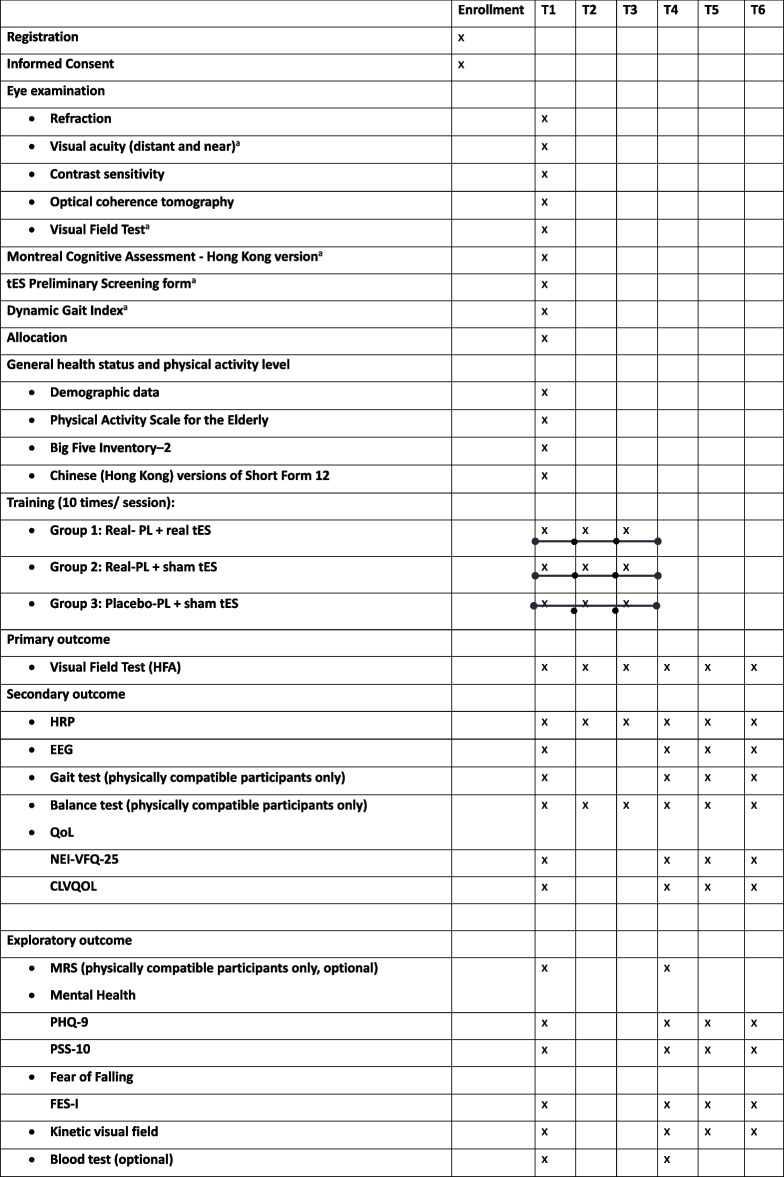
Timeline

*CLVQOL* Chinese version of the Low Vision Quality of Life Questionnaire, *EEG* electroencephalography, *FES-I* Falls Efficacy Scale-International, *HRP* high-resolution perimetry, *MRS* magnetic resonance spectroscopy, *NEI-VFQ-25* National Eye Institute Visual Function Questionnaire, *PHQ-9* Patient Health Questionnaire-9, *PL* perceptual learning, *PSS-10* Perceived Stress Scale, *QoL* quality of life, *tES* transcranial electric stimulation

^a^Eligibility test

### Sample size {14}

The sample size calculation for linear multiple regression with three groups and six time points was performed using G*Power (version 3.1). According to the HRP results from phase 1 study [37] in the GREAT project (Cohen’s *f*^2^ = 0.1), a sample of 144 glaucoma patients will be needed to provide 80% power to detect a significant difference in visual field among three groups at the 2-tailed 0.05 alpha level, assuming a 20% dropout rate.

### Recruitment {15}

Participants are being recruited from various sources including the optometry clinic at The Hong Kong Polytechnic University, Grantham Hospital, the Hong Kong Society for the Blind, self-help groups for patients, as well as private optometry and ophthalmology clinics using posters. The recruitment period started in July 2023 and is expected to end in December 2026. Recruitment is facilitated by the strategically placing posters at these locations. Moreover, to broaden our reach of this project, we have also launched advertisements on digital platforms such as YouTube and Facebook. At the stage of recruitment, participants are provided with comprehensive information about the study. This includes the timeline of the study, a detailed walkthrough of all study procedures, potential risks involved, and the benefits associated with each intervention. This ensures that participants are well informed and can make decisions based on a thorough understanding of the study.

## Assignment of interventions: allocation

### Sequence generation {16a}

A set of random numbers are generated by computer and the simple random sampling method is used to allocate the eligible participants into 3 groups.

### Concealment mechanism {16b}

An independent staff member assigns each participant a randomization number. Randomization is the only role in this project.

### Implementation {16c}

The independent staff member keeps the group assignments in a table that cannot be accessed by other members of the research team until data collection is completed and all datasets have been finalized.

## Assignment of interventions: blinding

### Who will be blinded {17a}

All eligible participants and investigators involved in the intervention and assessment procedures are blinded.

### Procedure for unblinding if needed {17b}

Unblinding will only take place after data lock. The study’s statistician, who will be unblinded, will complete the primary and secondary analyses according to the pre-specified statistical analysis plan. Other members of the study team will be granted to the unblinded datasets only after the primary and secondary analyses have been completed.

## Data collection and management

### Plans for assessment and collection of outcomes {18a}

All registered participants are recorded on a password protected glaucoma participant list and scheduled for screening eye exam using Google Calendar/Teams. All questionnaires are completed using REDCap. Raw data from measurements, including HFA, HRP, EEG, balance test, gait test, blood test, and MRI, are backed up on the hard drive. The recording forms for these measures are initially collected on paper, then scanned to create e-form after the assessments are completed. All investigators receive training in participant’s recruitment, intervention, assessments, and data backup before they participate in the project to ensure the reliability and validity of data collection process.

### Plans to promote participant retention and complete follow-up {18b}

The study aims to explore an approach that could enhance the rehabilitation of peripheral vision. The contributions of participants are highly valued and greatly appreciated throughout the study. The results of all outcomes, especially those from eye exams and balance test, are promptly shared with participants to help them fully understand their current status. In addition, a transportation allowance is provided to participants who complete all training and assessment procedures.

### Data management {19}

Deidentified data are kept in a Microsoft Teams group and on a hard drive that is accessible only to research team members. An identification number is generated for each participant to match the corresponding data files.

### Confidentiality {27}

Any information that is obtained in this study about enrolled participants will be confidential. Any publication or other public distribution of the experimental results will not include participants’ name. Raw data containing personal information will be destroyed upon the completion of this project. Research records are securely stored and only accessible for researchers. The Institutional Review Board of The Hong Kong Polytechnic University and University of Hong Kong/Hospital Authority Hong Kong West Cluster will also have access to records for the purpose of ethics review.

### Plans for collection, laboratory evaluation, and storage of biological specimens for genetic or molecular analysis in this trial/future use {33}

Blood samples are obtained from participants after obtaining informed consent. The venipuncture method is used, employing a sterile needle and syringe in conjunction with the vacutainer blood collection system. A volume of 6 mL of blood is collected from the antecubital vein into Greiner Bio-One 6 mL Vacuette Tubes. The collected blood is then allowed to clot undisturbed at ambient temperature for a duration of 30 to 60 min. To separate the serum from other blood constituents, the clotted blood is centrifuged at a force of 3000 × g and a temperature of 4 °C for 20 min. The resulting serum is transferred to a labeled 1 mL Eppendorf tube. To preserve the integrity of the serum samples, they are stored at a temperature of − 30 °C for further analysis.

## Statistical methods

### Statistical methods for primary and secondary outcomes {20a}

At the conclusion of this project, we will perform statistical analysis based on the intention-to-treat (ITT) principle. For the primary outcome, the differences of MD measured using the 24–2 SITA HFA will serve as an indicator of the effectiveness of interventions. Among the three groups, the intervention group showing the greatest improvement in MD will signify the strongest impact of rehabilitation. Additionally, changes in MD across six time points (baseline, interim 1, interim 2, post-intervention, 1-month post-intervention, and 2-month post-intervention) will reveal the dose-response relationship and recovery duration.

To address inter-individual variability, we will employ a linear mixed model with intervention type and time point as the fixed effect, and baseline MD value as the covariate. A dummy-coding scheme with the placebo PL + sham tES condition as the reference level will be utilized. Initially, a full model will be fitted, and if convergence issues or overfitting arise, adjustments will be made to the random intercept and slope. Model comparison will be conducted using a likelihood-ratio test to evaluate the adequacy of the current model relative to alternative models without the fixed effect. The level of statistical significance for analysis is set at a two-sided *p* < 0.05.

To address the potential attrition and missing data due to participant drop-out, we will conduct sensitivity analyses using multiple imputation methods. Specifically, we will employ the fully conditional specification (FCS) method. This method allows for the imputation of missing data for multiple variables with different distributions. By conducting these sensitivity analyses, we aim to assess the robustness of our primary analysis results to potential attrition bias and missing data. The multiple imputation approach will provide a principled way to handle missing data, ensuring that our conclusions are not unduly influenced by the potential non-random nature of missing data.

The analyses of secondary and exploratory outcomes will follow a similar approach to that of the primary outcome. A detailed statistical analysis plan for secondary and exploratory outcomes is being developed and will be reviewed of by the steering group prior to data analysis.

### Interim analyses {21b}

Not applicable. All data will be analyzed at the end of the experiment.

### Methods for additional analyses (e.g., subgroup analyses) {20b}

Not applicable. No additional analyses will be conducted.

### Methods in analysis to handle protocol non-adherence and any statistical methods to handle missing data {20c}

Missing data will be conducted by an intent to treat analysis (all randomized participants will be included with the last value carried forward), followed by a secondary “per-protocol” analysis (include only participants who followed all aspects of the protocol correctly).

### Plans to give access to the full protocol, participant-level data, and statistical code {31c}

The datasets analyzed during the current study and statistical code are available from the corresponding author on reasonable request, as is the full protocol.

## Oversight and monitoring

### Composition of the coordinating center and trial steering committee {5d}

The coordinating center is School of Optometry, The Hong Kong Polytechnic University, and the project steering committee consists core research team members including Prof. Allen Cheong, Prof. Ben Thompson, Prof. Bernhard Sabel, Prof. George Woo, and Dr. Dorita Chang. The monitoring committee and ethics committee are responsible for overseeing these procedures of this project. This includes the randomization of participants, ensuring the blindness of both participants and investigators, and maintaining the confidentiality of participants’ personal information.

### Composition of the data monitoring committee, its role and reporting structure {21a}

Not applicable. The intervention is non-invasive and has been proven safe.

### Adverse event reporting and harms {22}

Participants are closely monitored for any discomfort throughout the interventions and assessments. Investigators, who are also health care professionals and first aid qualified, will accompany and assist participants to ensure that they are not exposed to any hazards. If a research activity results in an injury, the laboratory is equipped with a first aid box and a telephone for emergency calls. Any participant discomfort is recorded in a serious adverse event form and simultaneously reported to the ethics committee and steering committee. These committees will determine if any further action is required, such as suspending data collection.

### Frequency and plans for auditing trial conduct {23}

The principal investigator monitors and audits trial conduct and data collection on a weekly basis. Researchers involved in various aspects of the study report their progress and challenges encountered during the experiment to the principal investigator each week. Besides, a data monitor is assigned to evaluate the integrity and quality of data every 2 weeks and provide feedback to the principal investigator accordingly.

### Plans for communicating important protocol amendments to relevant parties (e.g., trial participants, ethical committees) {25}

First, any proposed changes are discussed and approved by the steering committee. Second, any propose changes are communicated to the ethics committee, with a request for approval if necessary. Third, all study documentation and database entry forms are updated as necessary. Fourth, changes that affect data collection are communicated to the full research team and any current participants. Any changes to the participant’s study experience will also initiate a new consent process.

### Dissemination plans {31a}

The findings of this study will be disseminated in peer-reviewed scientific journals, research conferences, and seminars as part of continuing professional development. Also, the results of the intervention effect will be shared with all participants.

## Discussion

The progressive and irreversible nature of visual impairment caused by glaucoma has a significant profound impact on QoL and poses a serious public health concern. Consequently, there is an urgent need for novel rehabilitation methods that can enhance functional vision and improve the overall well-being of glaucoma patients.

The GREAT study aims to investigate the individual and combined effects of tES and PL on visual field, functional performances, brain activity, and various patient-reported outcomes in glaucoma patients. Importantly, this study examines the dose-response relationship and follow-up of any treatment effects, providing insights into the optimal dosage and duration of these interventions. Furthermore, the study monitors changes in brain activity (as measured by EEG), the neurotransmitter metabolites (as measured by MRS), and biomarkers (as measured by serum analysis) following the intervention. This comprehensive approach aids in understanding the underlying mechanisms and neurophysiological changes associated with the interventions, moving beyond the traditional focus on visual functions such as visual acuity and visual field. Additionally, the study assesses the impact of the intervention on real-world activities through gait and balance tests, as well as questionnaires evaluating mental health, QoL, and fear of falling. This holistic assessment is crucial, as the ultimate goal of vision rehabilitation is to improve functional independence and overall well-being for glaucoma patients.

While the study design is robust, there are several limitations that warrant acknowledgment. The repetitive assessments may induce learning effects across all measured outcomes, potentially limiting the ability to differentiate between true treatment effects and the effects of repeated testing. In addition, the long-term nature of the training sessions poses challenges for both participants and research staff, with fatigue and potential drop-out rates being realistic concerns in this large-scale clinical trial. Despite these limitations, it is important to recognize that if the treatment’s effectiveness is evident in the results, it could yield significant long-term benefits for glaucoma patients. Successful outcomes from this study could pave the way for the integration of tES and PL into standard vision rehabilitation protocols, potentially improving the QoL and functional independence for individuals affected by glaucoma.

## Trial status

Protocol version number and date: Version 2, 28th June 2024.

Date recruitment: Recruitment started in July 2023 and will be completed in December 2026.

### Supplementary Information

Supplementary Material 1. Appendix 1.

## Data Availability

Upon the completion of the project, the data and materials associated with it can be made available by the corresponding author upon a reasonable request.

## Abbreviations

BCVA: Best-corrected distance visual acuity
BDNF: Brain-derived neurotrophic factor
CLVQOL: Chinese version of the Low Vision Quality of Life Questionnaire
CS: Contrast sensitivity
EEG: Electroencephalography
ELISA: Enzyme-linked immunosorbent assay
ERP: Event-related potential
ETDRS: Early Treatment of Diabetic Retinopathy Study
FCS: Fully conditional specification
FES-I: Falls Efficacy Scale-International
GABA: Gamma-aminobutyric acid
Glx: Glutamate/glutamine
GREAT: Glaucoma Rehabilitation using ElectricAI Transcranial Stimulation
HFA: Humphrey visual field analyzer
HK-MoCA: Montreal Cognitive Assessment—Hong Kong version
HRP: High-resolution perimetry
ITT: Intention-to-treat
MD: Mean deviation
mfVEP: Multifocal visual evoked potential
MRI: Magnetic resonance imaging
MRS: Magnetic resonance spectroscopy
NEI-VEQ-25: National Eye Institute 25-Item Visual Function Questionnaire
OCT: Optical coherence tomography
PCR: Polymerase chain reaction
PHQ-9: Patient Health Questionnaire-9
PL: Perceptual learning
PSD: Pattern standard deviation
PSS-10: Perceived Stress Scale-10
QoL: Quality of life
RNFL: Retinal nerve fiber layer thickness
ROI: Region of interest
SITA: Swedish interactive threshold algorithm
SNR: Signal-to-noise ratio
SSVEP: Steady-state visual evoked potential experiment
tDCS: Transcranial direct stimulation
tES: Transcranial electrical stimulation
VEP: Standard visual evoked potentials
VFI: Visual field index
VOI: Volume of interest
