# Supplementary material for: Glaucoma Rehabilitation using ElectricAI Transcranial Stimulation (GREAT)—study protocol for randomized controlled trial using combined perceptual learning and transcranial electrical stimulation for vision enhancement
Source: Trials. 2024 Jul 22;25:501. doi: 10.1186/s13063-024-08314-3 (PMC11264395; doi:10.1186/s13063-024-08314-3)
Supplement: Supplementary file 1 — Supplementary Material 1. Appendix 1. [file 13063_2024_8314_MOESM1_ESM.docx]

**SUPPLEMENTARY MATERIALS**

**Appendix 1:**

| **Cycle** | **Gait measures** | | **Definition** |
| --- | --- | --- | --- |
| Cycle 1  (Preparation） • No obstacle • No visual task  Cycle 3 (Visual Searching) • No obstacle • Visual task  One cycle: • Divided by the cycle of non-dominant leg  • Gait between the two consecutive heel strikes) | Spatial-temporal parameters: (Dominant leg） | Walking Speed (m/s) | The distance traveled divided by ambulation time |
|  |  | Stride Length (mm) | The distance between the heel strike of two consecutive footfalls of the dominant leg |
|  |  | Step Width (mm) | The distance measured between the line of progression of the left foot and the line of progression of the right foot. |
|  | Kinematic parameters: (Dominant leg） | Hip Flexion/extension (degree) | The minimum/ maximum angle of hip movement |
|  |  | Knee Flexion/extension (degree) | The minimum/ maximum angle of knee movement |
|  |  | Ankle Flexion/extension (degree) | The minimum/ maximum angle of ankle movement |
|  |  | Head Flexion/Extension (degree) | The minimum/ maximum angle changes of head movement after comparing with the mean angle of the same patient |
|  |  | Response to the visual task | The number of right answers reported by patients/ number of right answers |
| Cycle 2  (Obstacle-crossing) • Obstacle • Visual task (searching) | Spatial-temporal parameters and  Kinematic parameters: | Same as above | |
|  | Crossing obstacle | Toe clearance (mm) | The toe height above the obstacle |
|  | Force plate | Sway of CoP on AP and ML directions (mm) | The difference between maximum and minimum values on AP and ML direction. |

CoP: Center of pressure; AP: Anterior-posterior; ML: Medial-lateral
